# Investigation of c-KIT and Ki67 expression in normal, preneoplastic and neoplastic canine prostate

**DOI:** 10.1186/s12917-017-1304-0

**Published:** 2017-12-06

**Authors:** Carlos Eduardo Fonseca-Alves, Priscilla Emiko Kobayashi, Chiara Palmieri, Renée Laufer-Amorim

**Affiliations:** 1 Department of Veterinary Clinic, School of Veterinary Medicine and Animal Science, Univ. Estadual Paulista – UNESP, Rua Professor Dr Walter Maurício Correa, s/n, Unesp/Campus de Botucatu, Mail box- 560, Botucatu, SP 18618-681 Brazil; 2 School of Veterinary Science, The University of Queensland, Gatton Campus, Gatton, Queensland Australia

**Keywords:** CD117 antigen, Dog, Prostatic cancer, Immunohistochemistry, Western blotting

## Abstract

**Background:**

c-KIT expression has been related to bone metastasis in human prostate cancer, but whether c-KIT expression can be similarly classified in canine prostatic tissue is unknown. This study assessed c-KIT and Ki67 expression in canine prostate cancer (PC). c-KIT gene and protein expression and Ki67 expression were evaluated in forty-four canine prostatic tissues by immunohistochemistry, RT-qPCR and western blot. Additionally, we have investigated c-KIT protein expression by immunoblotting in two primary canine prostate cancer cell lines.

**Results:**

Eleven normal prostates, 12 proliferative inflammatory atrophy (PIA) prostates, 18 PC, 3 metastatic lesions and two prostate cancer cell cultures (PC1 and PC2) were analysed. The prostatic tissue exhibited varying degrees of membranous, cytoplasmic or membranous/cytoplasmic c-KIT staining. Four normal prostates, 4 PIA and 5 prostatic carcinomas showed positive c-KIT expression. No c-KIT immunoexpression was observed in metastases. Canine prostate cancer and PIA samples contained a higher number of Ki67-positive cells compared to normal samples. The median relative quantification (RQ) for c-KIT expression in normal, PIA and prostate cancer and metastatic samples were 0.6 (0.1-2.5), 0.7 (0.09-2.1), 0.7 (0.09-5.1) and 0.1 (0.07-0.6), respectively. A positive correlation between the number of Ki67-positive cells and c-KIT transcript levels was observed in prostate cancer samples. In the cell line, PC1 was negative for c-KIT protein expression, while PC2 was weakly positive.

**Conclusion:**

The present study identified a strong correlation between c-KIT expression and proliferative index, suggesting that c-KIT may influence cell proliferation. Therefore, c-KIT heterogeneous protein expression among the samples (five positive and thirteen negative prostate cancer samples) indicates a personalized approach for canine prostate cancer.

**Electronic supplementary material:**

The online version of this article (10.1186/s12917-017-1304-0) contains supplementary material, which is available to authorized users.

## Background

Prostate cancer (PC) in dogs is associated with aggressive tumour behaviour, high metastatic rate and poor prognosis [1]. Bone and lungs are the most common metastatic sites, and 80% of patients show distant metastases at the time of diagnosis [2–4]. Despite the devastation of this disease, few treatment options are currently available for PC-affected dogs [5, 6]. Radiation therapy and non-steroidal anti-inflammatory drugs are considered the best options for metastatic PC [7]. Photodynamic therapy [8], targeted radioiodine imaging [9] and radiolabelled monoclonal antibodies [10] have been experimentally used in pre-clinical canine models of human prostate cancer, but their clinical efficacy is currently unknown. Other targeted therapies have been evaluated, such as inhibition of the c-KIT receptor using toceranib [11, 12]. In fact, recent studies have demonstrated the role of c-KIT in human PC development [13] and metastasis [14].

The proto-oncogene c-KIT belongs to the class III receptor tyrosine kinase family, and its ligand (stem cell factor protein) represents the mast cell growth factor in humans and dogs [15, 16]. In xenografts models of human PC with low c-KIT expression, the receptor is upregulated during cancer progression through production of stem cell factor in the bone microenvironment [14]. Thus, osteoclasts produce stem cell factor, stimulating PC cells to increase c-KIT expression and, subsequently, their migration from the primary tumour to bones [14]. Inhibition of c-KIT using lentiviral short hairpin RNA in human PC cell lines reduced tumour growth and increased the incidence of metastasis, suggesting a key role for c-KIT in intraosseous tumour growth in xenografts model [17].

Moreover, c-KIT has been hypothesized to be involved in neoplastic cell proliferation. In canine mammary tumours, a positive correlation between c-KIT and Ki67 expression has been demonstrated [18]. Brunettti et al. [18] observed a correlation between c-KIT expression and Ki67 immunoexpression, suggesting a correlation between the presence of c-KIT receptor and proliferative activity. These authors indicated that membranous or cytoplasmic immunolocalization promotes proliferation in canine malignant tissue [18]. Our research team evaluated double immunohistochemical expression of c-KIT/Ki67 in canine mast cell tumours [19] and identified a strong correlation between the co-expression of c-KIT and Ki67 with survival time. Thus, patients showing positive immunostaining against both markers (c-KIT/Ki67) experienced decreased survival time [20]. c-KIT expression has been studied in different canine tumours, including mast cell tumours [20], mammary tumours [18], seminomas [19], liposarcomas [19] and gastrointestinal stromal tumours [19]. To the best of our knowledge there is no information about c-KIT expression in canine PC tissue.

Therefore, this study aimed to evaluate c-KIT gene and protein expression in normal prostate, canine preneoplastic lesions (proliferative inflammatory atrophy – [PIA]), PC and its metastasis and correlation with Ki-67 expression and other clinical and biological parameters. Additionally, we investigated c-KIT protein expression in two canine PC primary cell lines.

## Methods

### Case selection

Forty-four formalin-fixed paraffin-embedded (FFPE) and twelve frozen canine prostatic tissues were selected from the archives of the Veterinary Pathology Service (Univ. Estadual Paulista-UNESP) from 2011 to 2015. Tumour samples were obtained from PC-affected dogs during prostatectomy, surgery or necropsy. Normal prostates and PIA lesions were collected during necropsy from animals that died causes not related to prostatic disease. For western blot analysis, tissue samples were collected and immediately frozen in liquid nitrogen.

All samples were from intact adult dogs. We selected 11 normal prostates, 12 PIA samples, 18 PC and three metastases. The metastatic samples were from three different patients (PC3, PC10 and PC13 – Table 1). The Gleason score of each PC was determined according to Palmieri and Grieco [21], and the histological subtypes were evaluated according to Palmieri et al. [22]. Only PC samples with negative Uproplakin III (UPIII) antibody staining were used. UPIII staining was performed according to Lai et al. [23].

**Table 1:** c-KIT and Ki67 expression in normal prostates and canine prostatic lesions

c-KIT Expression							
	c-KIT Staining		Distribution			Ki67	c-KIT
	Positive	Negative	Membranous	Cytoplasmic	Membr + Cytopl	Median of positive cells	Median of RQ
Normal (*n* = 11)	4	7	2	1	1	1	0.6
PIA (*n* = 12)	4	8	0	4	0	54.5	0.7
PC (*n* = 18)	5	13	0	2	3	366	0.7
Metastasis (*n* = 3)	0	3	0	0	0	578	0.2

*PIA* Proliferative Inflammatory Atrophy, *PC* prostate cancer, *Membr* membranous, *Cytopl* cytoplasmic

### Clinical data

Medical records were assessed to obtain clinical information, treatment modalities, treatment response and outcome for each patient. Clinical data was available for 14 out of 18 PC-affected patients (Additional file 1: Table S1). Radical prostatectomy was the primary treatment for animals with non-metastatic PC (2/14). Three patients (3/14) received metronomic chemotherapy, two patients (2/14) received piroxicam, while one patient (1/14) did not receive any treatment. Metronomic chemotherapy was administered according to Fonseca-Alves et al. [5].

### c-KIT and Ki67 protein expression

Slide sections (4 μm) were dewaxed in xylene and rehydrated in ethanol. For antigen retrieval, slides were incubated with citrate buffer (pH 6.0) in a pressure cooker (Pascal®; Dako, Carpinteria, CA, USA) followed by treatment with freshly prepared 3% hydrogen peroxide (code: 2081, Dinamica, Diadema, Brazil) in methanol (code: 956, Dinamica, Diadema, Brazil) for 20 min to inhibit endogenous peroxidase activity and further washed in Tris-buffered saline. Slides were incubated with the following primary antibodies overnight at 4 °C: anti-Ki67 (code: M7240, Monoclonal, mouse anti-human, Clone MIB1, Dako, 1:50) and anti-CD117/c-KIT (code: A4502, Polyclonal, rabbit anti-human, Dako, 1:100). Both antibodies have been previously validated against canine tissue [24, 25]. A polymer, peroxidase-based system (code: K4061, Envision, Dako, Carpinteria, CA, USA) was subsequently applied, and 3′-diaminobenzidine tetrahydrochloride (code: K3468, DAB, Dako, Carpinteria, CA, USA) was used as a chromogen for 5 min, followed by Harris haematoxylin (code: 2072, Dinamica, Diadema, Brazil) counterstaining. Negative controls were treated the same except for replacing the primary antibody with Tris-buffered saline. A cutaneous canine mast cell tumour was used as a positive control.

Samples were evaluated according to the percentage of c-KIT-positive expression in neoplastic cells and then classified as c-KIT positive or negative. The following semi-quantitative scores were applied: 0 = negative, 1 = >0–10% positive cells, 2 = 11–40% positive cells, 3 = 41–70% positive cells and 4 = > 70% positive cells. Samples showing a percentage higher than 10% were classified as positive [8]. Distribution of the signal (membranous and/or cytoplasmic) was recorded. For Ki67 analysis, the number of positive neoplastic cells in 10 high power fields (HPF) was calculated.

### Primary cell culture

Primary cell lines were previously established in our laboratory. We cultured one c-KIT positive and one c-KIT negative PC (evaluated by immunohistochemistry). Canine PC cells (PC1 and PC2) were cultured in a PEGM™ prostatic medium (Lonza, Basel, Switzerland) at 37 °C in 5% CO2 in culture medium supplemented with 10% inactivated foetal bovine serum (FBS, HYCLONE, Waltham, MA, USA) and 100 U/mL of penicillin G and 100 mg/mL of streptomycin (SIGMA, Portland, OR, USA). Culture medium was discarded and replaced with fresh medium every 48 h. For protein and mRNA extraction, 10^4^ cells were cultured in 6-well plates (Inc., Corning, NY, USA) in triplicate. When cell cultures reached greater than 90% confluence, protein and mRNA were extracted.

### Protein quantification

Western blotting was performed on normal prostate (*n* = 6), PC samples (n = 6) and the two cell lines. A cutaneous mast cell tumour was used as positive control for c-KIT expression. Tissue samples were mechanically homogenized in 50 mM of Tris–HCl buffer, pH 7.5, 0.25% Triton X-100 and EDTA using the Polytron homogenizer (Kinematica, Lucerne, Switzerland) for 30 s at 4 °C. For both cell lines, RIPA Lysis Buffer (catalog: 89,901, Millipore Co., Bedford, MA, USA) was used for protein extraction following manufacture’s recommendation. Then, sample homogenates from prostatic tissue and cell lines were centrifuged. Proteins were extracted from the supernatant and quantified as described by Bradford (1976). Equal amounts of protein (70 μg) obtained from the samples were heated at 95 °C for 5 min in the sample loading buffer and were then subjected to SDS–PAGE separation or electrophoresis under reducing conditions and transferred to nitrocellulose membranes (code: N7892, Sigma Chemical Co., St. Louis, MO). Membranes were blocked with 6% skimmed milk in TBS-T (10 mM Tris–HCl pH 7.5, 150 mM NaCl, 0.1% Tween-20) for 4 h, and the anti-KIT antibody (code: A4502, polyclonal, rabbit-anti human, Dako – CA, USA; 1:300) was applied and incubated overnight. Anti-β-actin antibody (code: sc-1616, polyclonal, goat anti human, Santa Cruz Biotechnology, Santa Cruz, CA, USA; 1:1:000) was used as a positive control. After incubation with the corresponding horseradish peroxidase-conjugated secondary antibody, the blots were detected by means of chemiluminescence (code: RPN2235, Amersham ECL Select western blotting Detection Reagent, GE Healthcare). Protein bands were quantified by densitometry analysis and expressed as integrated optical density (IOD). c-KIT protein expression was normalised to β-actin. Normalised data are expressed as the mean with standard deviation.

### Gene expression

All FFPE prostatic samples were macrodissected using 16-gauge needles, and mRNA were extracted using a commercial RecoverAll™ Total Nucleic Acid Kit (code: AM1975, Ambion, Life Technologies, MA, USA) according to the manufacturer’s instructions. mRNA from cell lines was extracted using the RNeasy mini Kit (code: 74,104, Qiagen, Hilden, Germany) following the manufacturer’s recommendations. mRNA concentration was determined with a spectrophotometer (NanoDrop™, ND-8000, Thermo Scientific, MA, USA), while mRNA integrity was evaluated with a Bioanalyzer 2100 and an Agilent RNA 6000 Nano Kit (code: 5067-1511, Agilent Technologies, CA, USA). cDNA was synthesized in included in a final volume of 20 μL, with each reaction containing 1 μg of total RNA treated with DNAse I (code: 18,047,019, Life Technologies, Rockville, MD, USA), 200 U of Super Script III reverse transcriptase (code: 18,080,044, Life Technologies, Rockville, MD, USA), 4 μL of 5X Super Script First-Strand Buffer, 1 μL of each dNTP at 10 mM (code: 18,427,088, Life Technologies, Rockville, MD, USA), 1 μL of Oligo-(dT)18 (500 ng/μL) (code: 8,418,012, Life Technologies, Rockville, MD, USA), 1 μL of random hexamers (100 ng/μL) (code: N8080127, Life Technologies, Rockville, MD, USA), and 1 μL of 0.1 M DTT (code: R0861, Life Technologies, Rockville, MD, USA). Reverse transcription was performed for 60 min at 50 °C, and the enzyme was subsequently inactivated for 15 min at 70 °C. cDNA was stored at −80 °C as described by Rivera-Calderón et al. [26].

RT-qPCR for c-KIT (Forward: 5′-CCAGTGTGTGGTTGCAGGAT-3′ and Reverse: 5′-CTCAGCTCCTGGACAGAAATACC-3′) and the endogenous genes *HPRT* (Forward: 5′-AGCTTGCTGGTGAAAAGGAC-3′ and Reverse: 5′-TTATAGTCAAGGGCATATCC-3′), *ACTB* (Forward: 5′-GGCATCCTGACCCTCAAGTA-3′ and Reverse: 5′-CTTCTCCATGTCGTCCCAGT-3′) and *RPS5* (Forward: 5′-TCACTGGTGAGAACCCCCT-3′ and Reverse: 5′-CCTGATTCACACGGCGTAG-3′) was conducted in a total volume of 10 μL containing Power SYBR Green PCR Master Mix (Applied Biosystems; Foster City, CA, USA), 1 μL of cDNA (1:10) and 0.3 μL of each primer. Reactions were performed in triplicate in 384-well plates using QuantStudio 12 K Flex Thermal Cycler equipment (Applied Biosystems; Foster City, CA, USA). A dissociation curve was included in all experiments to determine the PCR product specificity. Relative gene expression was quantified using the 2^-ΔΔCT^ method.

### Statistical analysis

All groups (normal, PIA, PC and metastases) were evaluated as the median, and an analysis of variance (ANOVA) was used to evaluate any difference among the groups. Data are presented as the mean ± SD. A t test was used to verify the significant difference in Ki67 expression and c-KIT gene expression. The survival curve was calculated only for PCs using the Kaplan-Meier method, and the statistical significance was determined using a log-rank test. The overall survival was defined as the period (in months) between the date of surgery and death. We evaluated the overall survival of all patients according to the chemotherapy protocol, c-KIT expression (c-KIT transcript level and immunoexpression) and Ki67 expression (low Ki67 expression or high Ki67 expression). The Ki67-positive samples were categorized as low or high according to the median expression. A t test was used for western blotting analysis. A t test was applied to analyse the c-KIT transcript levels comparing two variables. *P* < 0.05 was considered significant for all variables. Analyses were performed using GraphPad Prism 5 (GraphPad Software Inc., La Jolla, CA).

## Results

### Clinical data

All clinical information about breed, age and metastatic history from the fourteen dogs with PC are shown in Additional file 1: Table S1. Based on clinical records, the outcome was identified in 8 out of 14 (57%) patients. The median survival time was 165 days (12 – 423 days). Despite the heterogeneous survival rate due to different treatments, the patients that received metronomic chemotherapy experienced the highest survival time (*p* = 0.01). There were no significant differences between survival time and c-KIT or Ki67 expression. Based on the Gleason score classification, 10 patients showed a tumour with a Gleason score 10 (10/14), two patients showed a Gleason score 6 (2/14), one patient presented a Gleason Score 8 (1/14) and the other patient showed a Gleason Score 9 (1/14). Seven patients (7/14) showed metastases, primarily to lungs and bones. Four patients with metastases (4/7) showed PC with a Gleason score 10, one patient (1/7) had Gleason score 8 and one had a Gleason score 6 (1/7).

### Histology and growth pattern

**Fig. 1:**
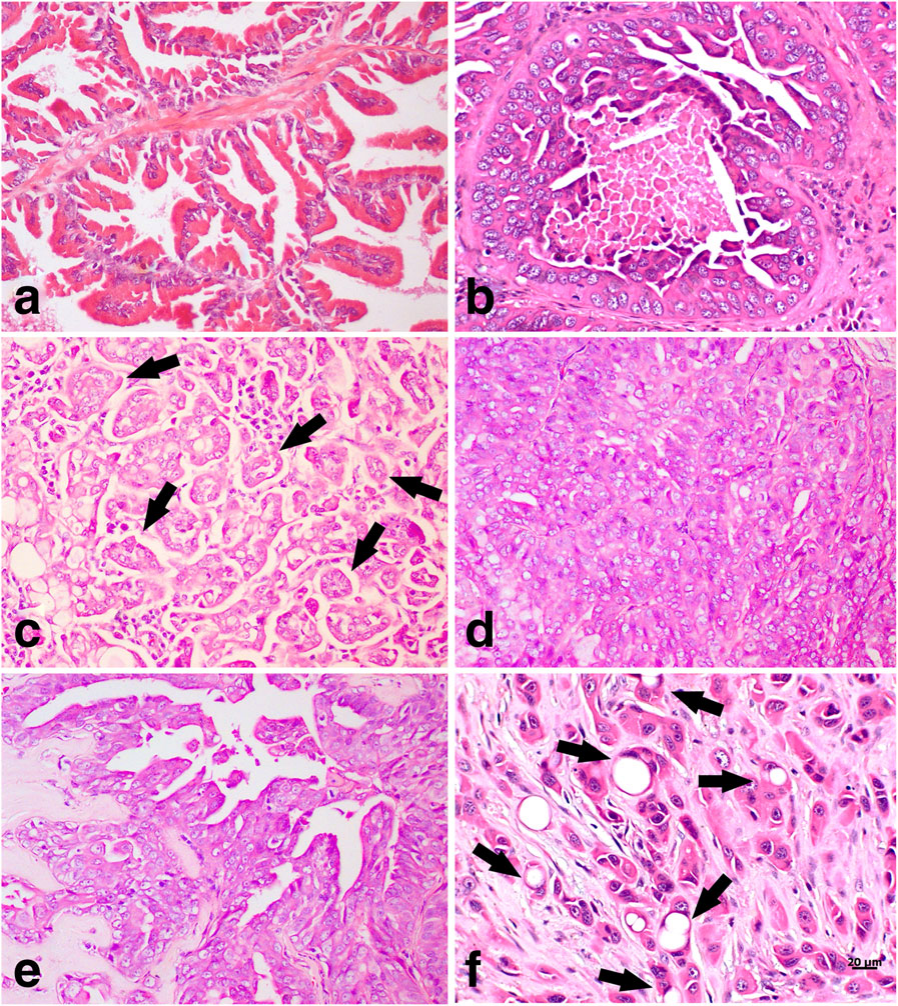
Histological evaluation of canine prostatic tissue. **a** Histological appearance of a normal prostate gland showing cuboid epithelial cells arranged in an acinar pattern. **b** Canine prostate cancer (PC) showing a cribriform pattern with central comedonecrosis (Gleason Score 10). **c** Canine PC presenting a small acinar pattern (black arrows) (Gleason score 6). **d** Histological appearance of a canine PC with a solid pattern (Gleason Score 10). **e** Canine PC showing a papillary pattern (Gleason Score 8). **f** Canine PC showing a signet ring (black arrows) histological pattern (Gleason Score 10)

The cribriform growth pattern was the most common histological subtype of PC (8/18), followed by the solid (5/18), small acinar (2/18), papillary (1/18) and signet ring (1/18). Only one sample (1/18) showed a mixed pattern (small acinar and cribriform). All histological subtypes are summarized in Additional file 1: Table S1 and Fig. 1. Regarding the co-existence of preneoplastic and neoplastic lesions, PIA lesions were observed surrounding carcinomas in 66.7% (12/18) of PC samples.

### c-KIT and Ki67 protein expression

**Fig. 2:**
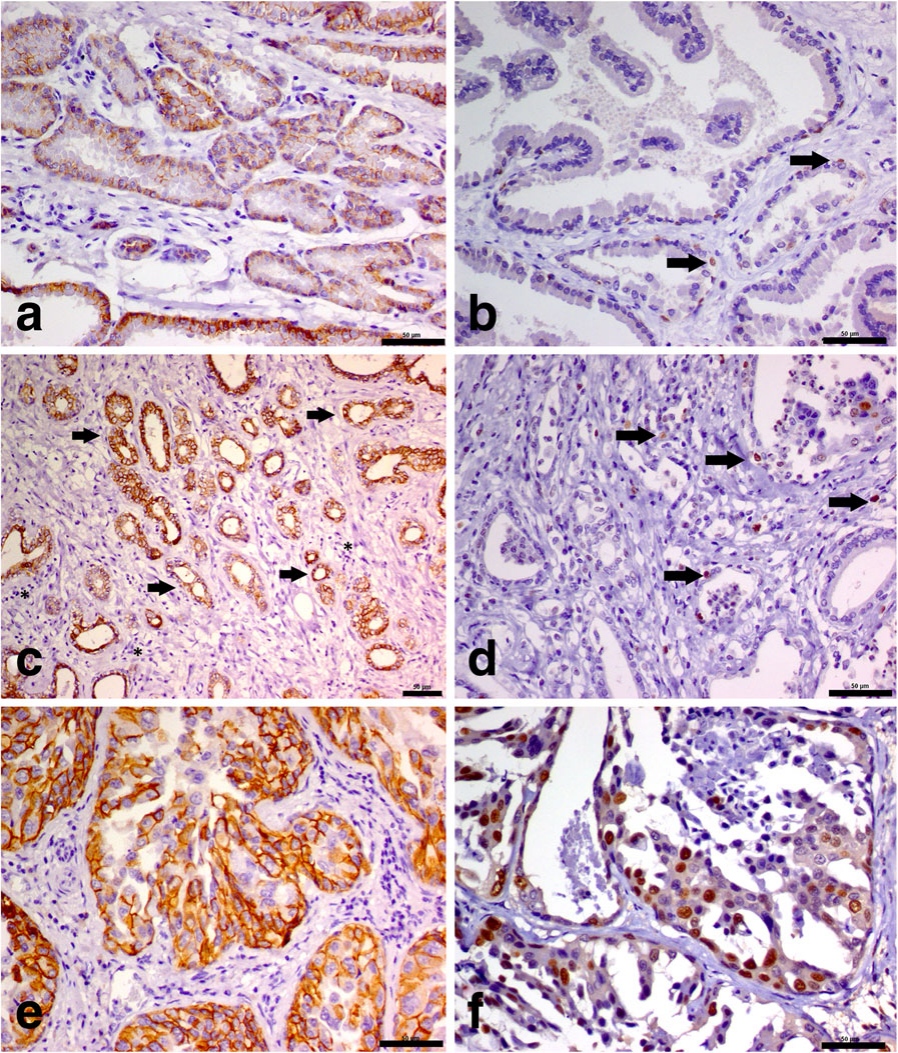
Immunohistochemical analysis of canine prostatic tissue for c-KIT and Ki67. **a** Normal canine prostate. Normal epithelial cells showing specific membranous staining (black arrow). **b** Ki67 expression in canine prostatic tissue. Arrows indicate positive nuclear expression in basal and stromal cells and no expression in luminal cells. **c** c-KIT expression in PIA lesion. Epithelial atrophic cells showed membranous and cytoplasmic c-KIT staining (arrows). **d** Ki67 expression in PIA lesion. There is positive nuclear expression in basal epithelial atrophic cells. **e** Canine prostate cancer. Neoplastic cells show membranous and cytoplasmic staining. Few c-KIT-positive stromal cells are observed. **f** Ki67 expression in canine prostate cancer. Note a high number of neoplastic cells showing nuclear expression. DAB chromogen, Harris haematoxylin counterstain. 200×

No correlation between histological subtype and c-KIT expression was identified. However, all PC samples positive for c-KIT expression showed a Gleason score 10. The immunohistochemical data are presented in Table 2. A total of 36.4% of normal samples (4/11) showed positive c-KIT staining (Fig. 2), and 33.3% (4/12) and 27.7% (5/18) of PIA and PC samples were c-KIT positive (Fig. 2). All metastases (3/3 – 100%) were c-KIT-negative. Regarding the semi-quantitative immunohistochemical analysis, 50% of c-KIT-positive normal samples (2/4) showed a score of 4 and 50% (2/4) showed a score of 3, while one c-KIT-positive PIA sample (1/4) had a score of 2 and the other three had scores of 3 (3/4). Two (2/5) c-KIT-positive PC samples had scores of 3, two (2/5) had scores of 2, and one (1/5) had a score of 4. Positive c-KIT staining was also observed in stromal cells (Fig. 2).

**Table 2:** Median of c-KIT gene expression and number of Ki67-positive cells in normal prostates and canine prostatic lesions according to c-KIT positive or negative immunostaining

	Normal c-KIT positive samples	Normal c-KIT negative samples	c-KIT positive PIA	c-KIT negative PIA	c-KIT positive PC	c-KIT negative PC	c-KIT negative metastasis
c-KIT gene Expression	1.6 (1.0-2.5)	0.6 (0.15-0.7)	1.3 (0.7-2.1)	0.2 (0.09-0.8)	1.5 (1.2-5.1)	0.5 (0.09-0.9)	0.1 (0.06-0.6)
Ki67-positive cells	2 (1-3)	0 (0-1)	75.5 (37-99)	49 (23-105)	645 (458-751)	301 (109-508)	578 (453-611)

PIA and PC samples contained higher numbers of Ki67-positive cells (Fig. 2) than did normal samples (Table 1). A progressive increase in the number of positive cells was observed in PIA, PC and metastatic samples (*p* < 0.0001). The mean number of positive cells in normal, PIA, PC and metastatic samples was 1.0, 54.5, 366 and 578, respectively (Table 1).

### Protein quantification

**Fig. 3:**
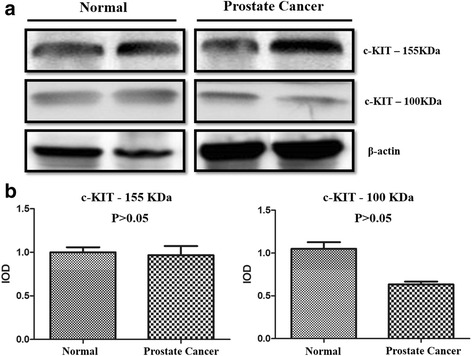
**a** Western blotting analysis of c-KIT expression in normal and prostate cancer (PC) samples. **b** No differences were observed between normal and PC samples

Two bands of 100 and 155 KDa were identified in canine prostatic tissue. Normal and PC samples showed a similar pattern of c-KIT expression with no significant difference between the two groups (Fig. 3). Three samples (3/6) with negative c-KIT immunoexpression were evaluated by western blotting, and we identified positive expression for both bands (100 and 155 KDa).

### Gene expression

The median relative quantification (RQ) for c-KIT expression in normal samples was 0.6. Normal samples showing positive c-KIT staining had a median of 1.6 RQ, and normal samples with a negative staining showed a median of 0.5 RQ. The median for PIA samples was 0.7 RQ for *c-KIT* expression, and positive samples showed a median of 1.3 RQ.

**Fig. 4:**
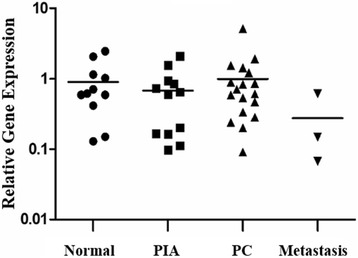
c-KIT transcript levels in normal prostate and canine prostatic lesions. There is a similar median of relative quantification among normal, proliferative inflammatory atrophy (PIA), prostate cancer (PC) and metastatic samples. In the PC group, pink triangles represent metastatic tumours

The median RQ for *c-KIT* expression in PC samples was 0.7 (Table 1). c-KIT-positive PCs had a median 1.5 RQ, and negative PCs showed a median 0.5 RQ. A lower median RQ for *c-KIT* expression was observed in the metastatic group (0.1) compared to normal samples (0.6). There were no significant differences among normal, PIA, PC and metastatic samples. All results are shown in Table 1, Table 2 and Fig. 4.

**Fig. 5:**
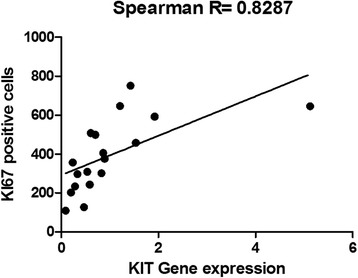
Spearman correlation between the number of Ki67-positive cells and c-KIT transcript levels in canine prostate cancer. There is a positive correlation (*R* = 0.8287) between the number of Ki67-positive cells and c-KIT transcript levels (prostate cancer samples with high number of Ki67-positive cells showed high c-KIT transcript levels)

A positive correlation between the number of Ki67-positive cells and c-KIT relative expression was identified (Spearman *R* = 0.8287; *P* < 0.0001). Tumours showing the highest *c-KIT* RQ had the highest number of Ki67-positive cells (Fig. 5).

### Primary cell culture

**Fig. 6:**
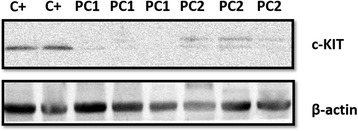
c-KIT protein expression in triplicate in canine cell lines. Positive control (canine mast cell tumour) exhibited strong c-KIT expression (100 KDa). The PC1 cell line was negative for c-KIT expression, and the PC2 cell line presented two weak bands

We grew both primary cell lines at passage 10. The PC1 cell line is from a primary tumour showing no c-KIT immunoexpression and presenting RQ 0.091 in RT-qPCR analysis. PC1 cells had no c-KIT protein expression (Fig. 6) and presented no detectable c-KIT transcript. The PC2 cell line is from a primary PC with c-KIT membranous and cytoplasmic immunoexpression and had 1.429 RQ by RT-qPCR analysis. The PC2 cells had very low c-KIT protein expression (Fig. 6) compared to controls (mast cell tumour) and showed 0.986 RQ by RT-qPCR.

## Discussion

We evaluated c-KIT protein and gene expression in canine prostatic tissue and found a small number of samples were positive for *c-KIT*. Our western blotting results identified positive protein expression in all samples analysed (6 normal and 6 PC), including three samples with KIT negative immunostaining. All prostatic samples contained transcript levels of c-KIT; however, only PCs with relative quantification higher than 1.211 (5/18) demonstrated positive c-KIT immunoexpression. Protein expression is regulated by a complex process involving DNA transcription, epigenetic modification and mRNA degradation [27], and protein expression is directly related to mRNA degradation and protein half-life [28]. Thus, changes and regulation of protein expression occur at multiple levels. One hypothesis for the differential c-KIT expression detected between different techniques is the difference in c-KIT protein and transcript stability in prostatic tissue.

On the other hand, some samples with detectible c-KIT transcripts had negative c-KIT immunostaining in epithelial cells and positive staining in stromal cells. The latter results were most likely associated with c-KIT-positive stromal cells observed by immunohistochemistry. This result may suggest that stromal cells play an important role in tissue microenvironment maintenance. The levels of protein and transcript detected by western blotting and RT-qPCR in samples showing c-KIT negative immunoexpression by epithelial cells could indicate that other cell types show c-KIT expression in these cases. These samples showed Ki67 expression only in basal and/or stromal cells, indicating a role for cell proliferation in c-KIT expression.

To evaluate c-KIT inhibition in vitro, we cultured two canine PC cell lines (one c-KIT positive and other c-KIT negative by immunohistochemistry), and evaluated protein expression by western blotting. PC1 cancer cells had no c-KIT protein expression, and PC2 cells showed very weak c-KIT expression. Moulay et al. [29] evaluated c-KIT transcript in five derived cancer cell lines and found only one line (DT08/40) was weakly positive. Detectable c-KIT transcript in the primary tumour from which PC1 cells were derived and the absence of transcript in the respective cell line could be related with expression of c-KIT by stromal cells, since the cell line contains only neoplastic epithelial cells.

c-KIT transcript levels were positively associated with the number of Ki67-positive cells. Therefore, a correlation between cell proliferation and c-KIT has been suggested [18. 20]. Thus, cells with high c-KIT transcript levels may have the ability to induce cell proliferation mediated by auto phosphorylation. Previously, we have demonstrated high number of c-KIT/Ki67 double-stained neoplastic cells in high-grade canine mast cell tumours [20]. These tumours showing double stained cells had internalization of the c-KIT receptor [20]. Mast cell tumours showing c-KIT I pattern presented few neoplastic mast cells with nuclear Ki67 and cytoplasmic c-KIT pattern. These findings suggest internalization of the c-KIT receptor during cell proliferation, at least in tumours showing a c-KIT I pattern [20].

No correlation has been demonstrated between c-KIT and Ki67 expression and histological subtype, while all PC samples with c-KIT positive staining had a Gleason score 10. In canine mammary gland tumours, Brunetti et al. [18] did not find any correlation between c-KIT expression and tumour histological type, invasiveness or type of mammary lesion. However, c-KIT expression was significantly associated with Ki67 index. The Ki67 value was higher in c-KIT positive tumours than in c-KIT negative tumours. The lack of clinical data in some patients has hindered the opportunity to identify a correlation between survival time and the prognostic markers under consideration. However, our results suggest a highest overall survival rate in patients receiving anti-inflammatory-based protocols.

Our results show differential c-KIT expression between primary PCs and metastatic lesions. Primary tumours showed a median c-KIT RQ of 0.7, while their corresponding metastases demonstrated lower transcript levels (median 0.1). Therefore, c-KIT may not be associated with the metastatic process in canine PCs. Interestingly, two (2/3) metastases were in bone, and human studies have shown that *c-KIT* signalling is associated with bone metastasis [14, 17]. Bone metastasis in human PC is strongly correlated with c-KIT and SCF signalling [14, 17]. A previous study using a xenograph model proposed that the bone microenvironment expresses SCF to induce expression of c-KIT by neoplastic epithelial cells, acting as an attractant for neoplastic cell migration [14]. It should be noted that this study has a limited number of samples, and there is a need to assess more samples from various breeds of dog and different age groups. More in-depth studies are needed to investigate the direct relationship between c-KIT and cell proliferation rate (Ki67).

Targeted drug therapy has been used in veterinary medicine for the treatment of canine PC. Controversial results have been obtained in canine PCs treated with toceranib, a multi targeted inhibitor with activity against c-KIT and other tyrosine kinases; stable disease for at least 6 weeks has been reported by Chon et al. [11], while partial response or progressive disease was reported by Pan et al. [12] However, our c-KIT results suggest that dogs with c-KIT-positive PC may benefit from this drug or other compounds with a specific activity against this receptor.

## Conclusions

The present study identified a strong correlation between c-KIT expression and high proliferative index, suggesting c-KIT may influence cell proliferation. We identified no relationship between c-KIT expression and metastatic tumours. Therefore, heterogeneous protein expression of c-KIT among samples (five positive and thirteen negative PC samples) indicates a personalized approach is needed for canine PC. Thus, each canine PC should be evaluated individually for more accurate patient care.

## Additional file

Additional file : Table S1. Clinical data and Gleason score of dogs with prostate cancer (DOCX 14 kb)

## Abbreviations

ANOVA: Analysis of variance
cDNA: Complementary deoxyribonucleic acid
FFPE: Formalin-fixed paraffin-embedded
PC: Prostate cancer
PIA: Proliferative inflammatory atrophy
RNA: Ribonucleic acid
UPIII: Uroplakin III
