# Supplementary material for: Investigation of c-KIT and Ki67 expression in normal, preneoplastic and neoplastic canine prostate
Source: BMC Vet Res. 2017 Dec 6;13:380. doi: 10.1186/s12917-017-1304-0 (PMC5718037; doi:10.1186/s12917-017-1304-0)
Supplement: Additional file : Table S1. — Clinical data and Gleason score of dogs with prostate cancer (DOCX 14 kb) [file 12917_2017_1304_MOESM1_ESM.docx]

Additional file 1: Table S1. Clinical data and Gleason score of dogs with prostate cancer.

|  |  |  |  |  |  |  |  |
| --- | --- | --- | --- | --- | --- | --- | --- |
| **Case*** | **Breed** | **Age** | **Metastases** | **Histological Pattern** | **Gleason score** | **Treatment** | **Outcome (days)** |
|  |  |  |  |  |  |  |  |
| PC 1 | Boxer | 14 | Lung and Bone | Cribriform | 10 | Piroxicam | 90 |
| PC 2 | MBD | 12 | No | Papillary | 8 | Piroxicam | 240 |
| PC 3 | Brazilian Mastiff | 8 | Bone | Solid | 10 | LDMT | 320 |
| PC 4 | MBD | 11 | No | Signet ring | 10 | N/A | N/A |
| PC 5 | MBD | 13 | Bones, Intestine, Lung | Small acinar + cribriform | 8 | NT | 12 |
| PC 6 | MBD | 15 | Bone | Cribriform | 10 | LDMT | 423 |
| PC 7 | American Cocker spaniel | 10 | No | Solid | 10 | RP | 32 |
| PC 8 | Teckel | 11 | No | Cribriform | 10 | N/A | N/A |
| PC 9 | Boxer | 12 | Bone, Lung | Cribriform | 10 | LDMT | 278 |
| PC 10 | MBD | 14 | Intestine | Solid | 10 | N/A | N/A |
| PC 11 | Poodle | 8 | No | Small acinar | 6 | RP | 45 |
| PC 12 | MBD | 9 | No | Cribriform | 10 | N/A | N/A |
| PC 13 | American Pitbull terrier | 10 | Lung | Small acinar | 6 | N/A | N/A |
| PC 14 | MBD | 13 | No | Cribriform | 10 | N/A | N/A |
| MBD: Mixed Breed dog; N/A Not Available; NT No Treatment; RP Radical Prostatectomy; LDMT Low-dose metronomic therapy; *No clinical information are available in four cases. | | | | | | | |
